# Gliadin Fragments and a Specific Gliadin 33-mer Peptide Close K_ATP_ Channels and Induce Insulin Secretion in INS-1E Cells and Rat Islets of Langerhans

**DOI:** 10.1371/journal.pone.0066474

**Published:** 2013-06-13

**Authors:** Morten Dall, Kirstine Calloe, Martin Haupt-Jorgensen, Jesper Larsen, Nicole Schmitt, Knud Josefsen, Karsten Buschard

**Affiliations:** 1 The Bartholin Institute, Rigshospitalet, Copenhagen, Denmark; 2 Danish National Research Foundation Centre for Cardiac Arrhythmia, Dept. of Biomedical Sciences, Faculty of Health and Medical Sciences, University of Copenhagen, Copenhagen, Denmark; La Jolla Institute for Allergy and Immunology, United States of America

## Abstract

In non-obese diabetic (NOD) mice, diabetes incidence is reduced by a gluten-free diet. Gluten peptides, such as the compound gliadin, can cross the intestinal barrier and may directly affect pancreatic beta cells. We investigated the effects of enzymatically-digested gliadin in NOD mice, INS-1E cells and rat islets. Six injections of gliadin digest in 6-week-old NOD mice did not affect diabetes development, but increased weight gain (20% increase by day 100). In INS-1E cells, incubation with gliadin digest induced a dose-dependent increase in insulin secretion, up to 2.5-fold after 24 hours. A similar effect was observed in isolated rat islets (1.6-fold increase). In INS-1E cells, diazoxide reduced the stimulatory effect of gliadin digest. Additionally, gliadin digest was shown to decrease current through K_ATP_-channels. A specific gliadin 33-mer had a similar effect, both on current and insulin secretion. Finally, INS-1E incubation with gliadin digest potentiated palmitate-induced insulin secretion by 13% compared to controls. Our data suggest that gliadin fragments may contribute to the beta-cell hyperactivity observed prior to the development of type 1 diabetes.

## Introduction

Gluten is a wheat protein that confers elasticity to white bread, and it is universally present in the western diet. Gluten consists of two families of prolamins, known as gliadin and glutenin. Gliadin is a strongly hydrophobic glycoprotein, with a very poor solubility. This severely limits its enzymatic degradation, which results in the persistence of gliadin fragments in the gut and intestine. This has been reported to initiate subclinical inflammation in the intestinal mucosa [1]. Up to 2% of Caucasians develop celiac disease, also known as gluten intolerance, which is an immune-mediated enteropathy. A variety of proline-rich, protease-resistant gliadin fragments are implicated in the pathogenesis of celiac disease, including a specific 33-mer peptide [2]. E*x vivo*, the 33-mer is transported into duodenal cells, where it escapes degradation [3]. A gliadin 19-mer has also been described as a gliadin digestion product, and transport of the 19-mer across the epithelial barrier is well-established in untreated celiac disease patients [3]. Non-degraded gliadin has been identified in breast milk of healthy mothers [4], providing evidence that gliadin can pass undigested from the intestine into the bloodstream. Patients with type 1 diabetes mellitus (T1D) have increased intestinal permeability [5], which suggests that gluten exposure may be increased in this group.

The association between gluten consumption and T1D is well established. We have shown that a gluten-free diet lowers diabetes incidence in non-obese diabetic (NOD) mice from 64% for chow fed controls, to 15% for the gluten-free group [6]. This can be further reduced to 6% by keeping the mother on a gluten-free diet during pregnancy [7]. A gluten-free diet is also protective in the BB (Biobreeding) rat, a rat model for T1D [8]. Emerging observations indicate that a gluten-free diet may protect against T1D in humans; thus, we have described a newly diagnosed T1D patient who has been prescribed a gluten-free diet, and has now been living free of insulin therapy for 36 months [9]. Furthermore, there is evidence of a decreased tolerance to gluten in T1D patients, since up to 10% of T1D patients have celiac disorders [1]. Interestingly, T1D most often develops prior to decreased gluten tolerance, which may illustrate the prophylactic effect of a gluten-free diet on the development of T1D [10]. The mechanism by which a gluten-free diet inhibits diabetes is yet unknown. In this study, we consider a possible mechanism underlying the protective effect of the gluten free diet by investigating gliadin's contribution to beta-cell activation.

## Materials and Methods

### Ethics

Animal experiments were conducted at the University of Copenhagen in accordance with the revised Council of Europe Convention ETS 123, the revised EU directive 2010/63 and the Danish Proclamation of law on animal experimentation 1306 from 23.11.2007, and was approved specifically by the Danish Animal Experiments Inspectorate and the Animal Ethics Council (license no. 2010-561-1851, project no. P 924). Animals were under the supervision of veterinarians, and their well-being was monitored by the staff of the animal facility daily. Animals were fed an Altromin 1324 diet (Brogaarden, Lynge, Denmark) and had access to bottled tap water. All cages had aspen bedding, a cardboard house and a wooden chewing block. Lighting was present from 6.00 AM to 6.00 PM, and air was changed in the cage 10–12 times per hour. Diabetic animals were sacrificed by cervical dislocation

### Chemicals

Unless otherwise noted, chemicals were purchased from Sigma-Aldrich (St. Louis, Missouri) and cell culture materials from Greiner Cellstar (Radnor, Pennsylvania).

### Gliadin digestion

Gliadin and ovalbumin were digested as described [2], with the following modifications: 250 mg of protein was added to 2.5 ml of 0.1 M HCl, and pH was adjusted to 2.0. After addition of 2.5 mg pepsin (Fluka/Sigma-Aldrich), the mixture was incubated at 37°C for a minimum of 5 hours, until all gliadin had been dissolved. Following incubation, 500 µl of 50 mM phosphate buffer (pH 7.0) was added, and pH was titrated to 7.0 using 3 M NaOH. Trypsin (Fluka/Sigma-Aldrich), chymotrypsin (AppliChem, Darmstadt, Germany) and carboxypeptidase (1.6 mg of each), were added with 320 µg elastase (Fluka/Sigma-Aldrich), and the mixture was incubated at 37°C for 3 hours. The solution was heat-inactivated at 80°C for 5 min and centrifuged at 20.000× g for 20 min before sterile filtration (*in vivo*: 5 µm filter 17594K (Sartorius, Göttingen, Germany); *in vitro*: 0.2 µm mixed cellulose filter DG2M-110 (Spectrum, Rancho Dominguez, CA)). Aliquots were stored at −20°C. Protein concentration was determined by the BCA method (Pierce, Rockford, IL, USA). A Spectra/Por® Float-A-Lyzer® G2 dialyser device (MWCO 100–500 Da, Spectrum labs) was used to dialyze gliadin and a Microcon Centrifugal Filter Device (MWCO 3000 Da, Merck Millipore, Billerica, MA, USA) was used to make a high molecular gliadin digest. Endotoxin levels in gliadin digest and digestion enzymes were quantified using a LAL QCL-1000 assay (Lonza).

### Animal Experiments

Forty-five female NOD mice 6 weeks of age (Taconic, Hudson, New York) were acclimatized for one week prior to the experiments. The mice received intravenous injections with 0.15 ml PBS with 0, 4.5 or 450 µg digested gliadin six times over two weeks (3–4 days between injections). Twice a week, the mice were weighed and blood glucose levels were quantified using an Abott Freestyle Lite device (Abbot, Abbott Park, Illinois), using 1 µl of blood for quantification to minimize discomfort for the animals. After three subsequent measurements of a blood glucose concentration above 12 mM, the animals were considered diabetic and were sacrificed. To investigate the acute effect of gliadin injection, 12 10-week old offspring of NOD mice were fasted for 4 hours, and injected with either 450 µg gliadin digest or a corresponding volume of heat-inactivated digestion enzymes. Blood glucose levels were monitored over 3 hours, and 8 blood samples were taken from each mouse at various time points for insulin ELISA.

### Cell culture

INS-1E cells were kindly provided by P. Maechler from the University of Geneva, where the cell line was initially established [11]. Cells (passage 79–90) were grown in RPMI 1640 (Invitrogen, Carlsbad, California) supplemented with 10% FCS (Gibco, Invitrogen), 1% Na-pyruvate (Gibco, Invitrogen), 1% HEPES (Gibco, Invitrogen) and 50 µM mercaptoethanol. For experiments, 4×10^5^ cells/well were seeded in 12-well plates, and 2×10^4^ or 4×10^4^ cells/well were seeded in 96-well plates, dependent upon the assay. Medium was replaced with RPMI 1640 supplemented with 0.5% FCS and stimulants 24 hours later. Cells were incubated for 24 hours. Resazurin [12] dissolved in PBS (0.11 mg/ml) was added to cell medium for 2 hours at a concentration of 11 µg/ml, and resazurin conversion was detected as fluorescence. Cellular ATP levels were measured using an adenosine 5-triphosphate (ATP) bioluminescent somatic cell assay kit (Cat # FLASC). Fluorescence and luminescence were detected on a Fluoroscan Ascent TL (Thermo Fisher Scientific, Waltham, MA). In pre-incubation experiments, cells were exposed to 300 µg/ml gliadin digest in RPMI for 24 hours, then incubated in RPMI with 3 mM glucose and 0.5% FCS for 2 hours (also containing gliadin digest) and finally Ca-5 buffer [13] supplemented with 3 mM glucose for 30 min Stimulation was performed in Ca-5 buffer supplemented with 3 or 11 mM glucose. In relevant cases, the buffer also contained gliadin digest or palmitate. For palmitate stimulation, cells were pre-treated as above. Palmitic acid was added to 0.1 M NaOH for a final concentration of 100 mM. The mixture was heated to 70°C, and added to Ca-5 buffer to a final concentration of 500 µM. After 30 min the supernatant was harvested, centrifuged at 200× g for 5 min, and stored at −20°C before insulin ELISA (Mercodia, Uppsala, Sweden) was performed.

### Gliadin fragment experiments

The gliadin 19-mer and deamidated 33-mer (LGQQPFPPQQPYPQPQPF-OH and LQLQPFPQPELPYPQPELPYPQPELPYPQPQPF) were synthesised by Schafer-N (Copenhagen, Denmark), and purity was confirmed by HPLC analysis. Fragments were dissolved in RPMI 1640, supplemented with 0.5% FCS and 11 mM glucose. Solutions were sterile filtrated through a low-protein binding PVDF 0.22 µm Millex® filter (Millipore).

### Islet isolation and culture

Islets were isolated from male Lewis Rats (Charles River lab, Wilmington, MA, USA), using collagenase infusion via the pancreatic duct [14]. After isolation, islets from four rats were pooled and cultured in RPMI 1640 supplemented with 10% fetal calf serum at 37°C and 5% CO_2_ in 12-well plates. Prior to stimulation, islets were placed in RPMI 1640 containing 3 mM glucose, supplemented with 10% FCS for 24 hours. The medium was changed to RPMI 1640 with 3 mM glucose and 0.5% FCS, and after 2 hours, gliadin digest and/or glucose were added at relevant concentrations, and islets were incubated for 24 hours. Supernatants were stored for insulin ELISA measurements.

### siRNA downregulation

The following siRNAs were purchased from Ambion (Austin, TX): MyD88 siRNA: ID s141418, FFAR1 siRNA: ID s153613, Control siRNA: Silencer Select Negative Control No. 1 siRNA

For each well, 20 µmol of siRNA (stock: 20 µM) was transfected into cells with 2 µl of DharmaFECT2 (Thermo Scientific, Waltham, MA) and Optimem (Gibco, Invitrogen). One day following transfection, cells were stimulated with gliadin digest for 24 hours. Supernatant was harvested, and RNA was isolated using Trizol. RNA concentration was determined using a Nanodrop 1000 (Thermo Scientific). RNA was reversely transcribed to cDNA using qScript cDNA SuperMix (Quanta Biosciences, Gaithersburg, MD).

### Primers

Primers were designed using Primer-3 software and synthesised by TAGC (Copenhagen, Denmark). Sequences were as following:

Actin: 5′-AGC-CAT-GTA-CGT-AGC-CAT-CC-3′ and 5′-CTC-TCA-GCT-GTG-GTG-GTG-AA-3′. FFAR1: 5′-CAG-AGG-CTG-GGT-GGA-TAA-CA-3′ and 5′-AGC-CCA-CAT-AGC-AGA-AAG-CA-3′. MyD88: 5′-ATC-CCA-CTC-GCA-GTT-TGT-TG-3′ and 5′-CTC-CTG-TTT-CTG-CTG-GTT-GC-3′.

### qPCR

Quantitative PCR was carried out on a Lightcycler II (Roche, Penzberg, Germany), using SYBR II premix (Takara, Otsu, Japan). Standard curves for qPCR were made by dilution of specific PCR products. Specificity was confirmed by sequencing (GATC Biotech, Konstanz, Germany). qPCR was run using the following parameters: 95°C 10 sec, 45× (95°C 10 sec, 58°C 5 sec, 72°C 15 sec). Reaction specificity was determined by melting curve analysis. Data were analysed using the Lightcycler software. Expression was normalised to actin expression.

### Cell culture and transfections for electrophysiology

Human embryonic kidney (HEK-293) cells were cultured in Dulbecco's modified Eagle's medium (in-house, University of Copenhagen, Denmark) with 10% fetal calf serum (Gibco, Invitrogen) at 37°C in 5% CO_2_. Cells at 80% confluence were transfected with 0.5 µg hK_ir_6.2 (GenBank Acc.No. NM_000525) and 1.5 µg hSUR1 (NM_000352) in pCDNA3 using Lipofectamine and Plus reagent (Gibco, Invitrogen). Enhanced GFP was added for identification of transfected cells. Some cells were incubated overnight with 300 µg/ml gliadin digest, enzyme solution (for control), or 100 µM gliadin 19-mer or 33-mer added to the cell medium.

### Electrophysiology

Whole-cell currents were recorded 1–2 days post transfection using a MultiClamp 700B amplifier and MultiClamp Commander (Axon Instruments, Foster City, CA, USA). The cells were superfused with a solution containing (in mM): 138 NaCl, 5.6 KCl, 1.2 CaCl_2_, 2.6 MgCl_2_, 10 HEPES, 3 glucose, and adjusted to pH = 7.2 with NaOH. Patch pipettes were fabricated from borosilicate glass capillaries (Module Ohm, Herlev, Denmark) and had tip resistances between 1.5 and 2.0 MΩ when filled with pipette solution of the following composition (mM): 107 KCl, 2.0 MgCl_2_, 1.0 CaCl_2_, 10 NaCl, 10 HEPES and 10 EGTA, pH = 7.2 with KOH. The solutions were modified from [15] and all experiments were performed at room temperature (20–22°C). A 70–85% electronic compensation of series resistance was applied to the system. All analog signals were acquired at 10–50 kHz, filtered at 6 kHz and digitized with a Digidata 1440 converter (Axon Instruments).

### Statistics and data analysis

Data were analyzed using paired and unpaired t-tests, ANOVA and Mantel-Cox tests, which were applied with Graphpad Prism 5.0 software. A one sample t-test was used when groups were compared against a control group with a defined value of one. Unless otherwise is noted, values are given ±95% confidence interval. The following symbols were used for the figures: *: P<0.05, **: P<0.01, ***: P<0.001, relative to medium with 11 mM glucose unless otherwise noted.

## Results

### Gliadin digest induces weight gain in NOD mice and acutely lowers blood glucose levels

**Figure 1:**
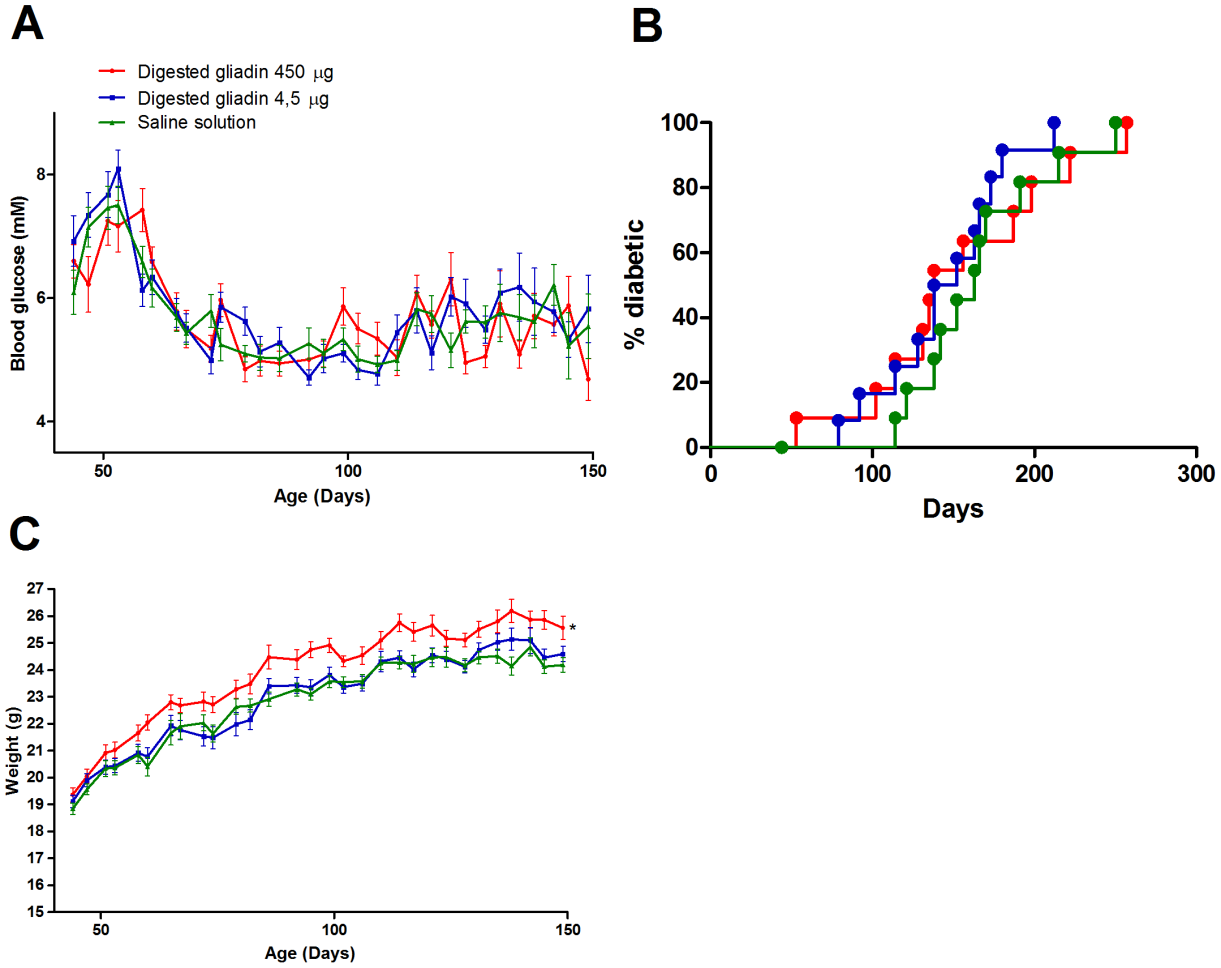
Gliadin digest increases weight in NOD mice. NOD mice were injected with gliadin digest (blue: 4.5 µg, red: 450 µg, green: controls, displayed as mean±SEM) six times over two weeks. Blood glucose level and weight were measured twice a week. A. Comparison of blood glucose levels. No significant differences were observed between the groups (P = 0.84, n = 15). B. Diabetes incidence. No differences were observed between groups (P = 0.53, n = 15, Mantel-Cox). C. Weight. There was a significant weight increase in mice injected with the 450 µg gliadin digest doses (average 24.9±0.9 g at day 100) compared to controls (average 23.6±0.7 g at day 100) (P<0.0001, n = 15).

In NOD mice, we observed no change in blood glucose levels (Fig. 1A) or diabetes incidence (Fig. 1B) after six gliadin digest injections from the mice were 6 weeks old. At 99 days of age, the mice receiving the 450 µg gliadin digest doses weighed significantly more than controls (24.9±0.9 g and 23.6±0.7 g, respectively, P<0.0001, n = 15, Fig. 1C), corresponding to a 20% increase in weight gain from the beginning of experiment (5.6±0.8 g for mice injected with 450 µg gliadin vs. 4.7±0.5 g for controls, P<0.0001). Weight increased shortly after the injections and persisted until after the mice were 200 days old, by which point a majority of the mice had developed diabetes and were euthanized. A single injection of gliadin digest did not significantly change blood glucose levels or insulin levels for 3 hours following the injection (data not shown).

### Gliadin digest and gliadin 33-mer increases insulin secretion in INS-1E rat insulinoma cells

**Figure 2:**
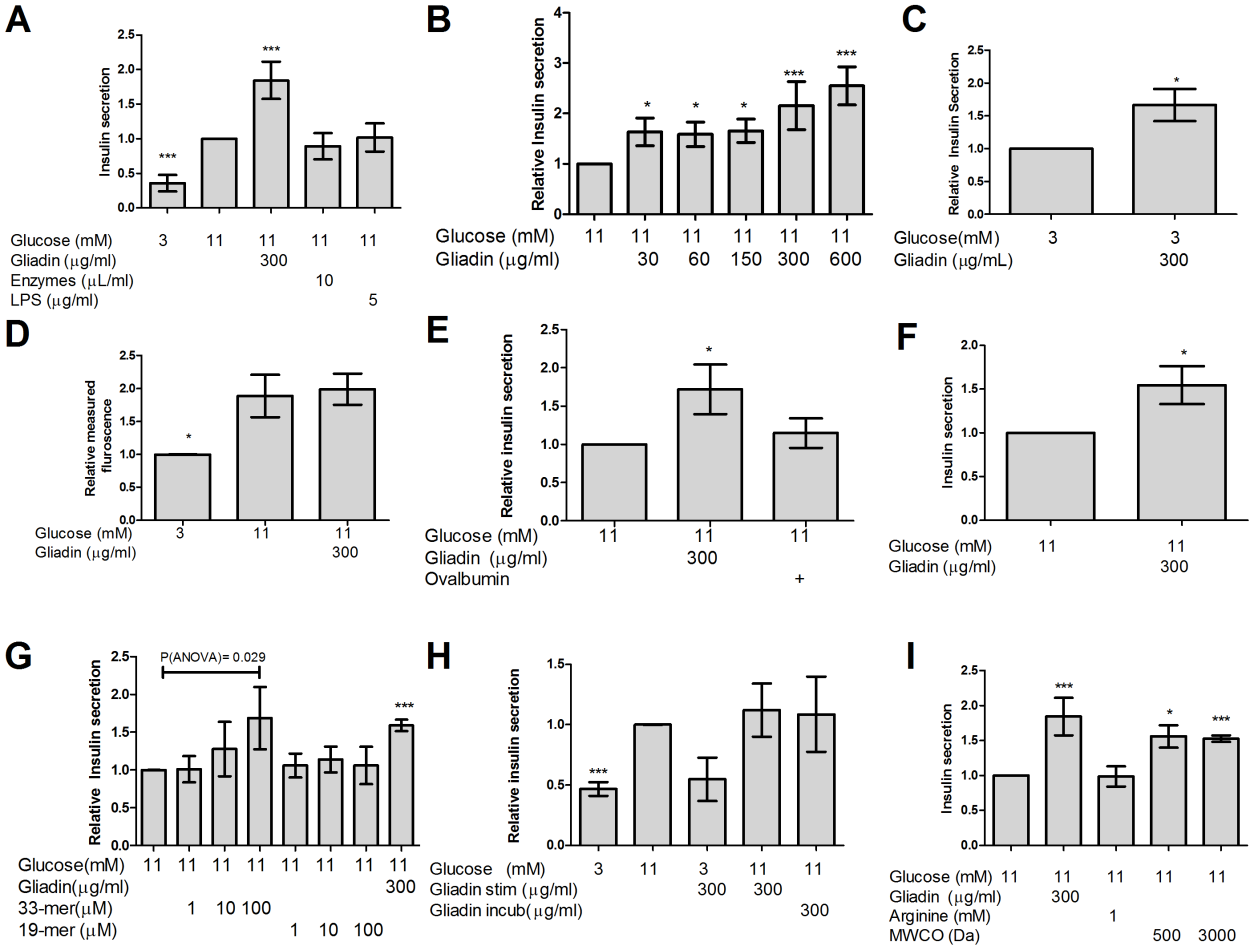
Gliadin digest and a gliadin 33-mer increases insulin secretion in INS-1E cells and rat islets during 24 hours of stimulation. A. Gliadin digest significantly increased insulin secretion 1.8±0.3fold (P<0.001, n = 6). No effect was observed from heat-inactivated digestion enzymes (P = 0.27, n = 5) or lipopolysaccharide (P = 0.46, n = 4). B. In INS-1E cells, insulin release increased dose-dependently during stimulation for 24 hours with increasing concentrations of gliadin-digest, as compared to glucose stimulation alone (1.5±0.29 fold increase for 30 µg/ml to 2.5±0.4 fold for 600 µg/ml, P<0.0001, n = 6). C. Addition of gliadin digest to cells in low glucose increased insulin secretion by 50% (1.5±0.4X, P = 0.01, n = 4). D. Quantification of live cells with resazurin. Data were normalized relative to the number of cells in 3 mM glucose after 24 hours. No difference was observed between the mass of cells stimulated in 11 mM glucose and that of cells treated with 11 mM glucose and gliadin digest (P = 0.626, n = 4). E. Incubation with 11 mM glucose and enzymatically digested ovalbumin for 24 hours did not increase the insulin secretion significantly (P = 0.22, n = 4). Gliadin digest significantly increased insulin secretion compared to ovalbumin (1.57±0.46x, P = 0.034, n = 3). F. Gliadin digest-stimulated insulin secretion in rat islets of Langerhans was significantly increased up to 1.55±0.35 times compared to controls (P = 0.015, n = 4). G. Stimulation of INS-1E cells with increasing amounts of 33-mer in 11 mM glucose, resulted in up to 1.7 fold dose-dependent increase in insulin secretion compared to the control group (P = 0.03, n = 4). The 19-mer had no effect on insulin secretion at any concentration assayed (P = 0.98, n = 4). H. During 30 min stimulation, INS-1E cells stimulated with 3 mM glucose secreted significantly less insulin than cells stimulated with 11 mM glucose (0.47±0.09, P<0.001, n = 4). Co-incubation with gliadin digest did not increase insulin secretion (P = 0.35, n = 4). Incubation with gliadin digest for 24 hours prior to glucose stimulation did not affect insulin secretion (P = 0.62, n = 4). I. Arginine (1 mM) had no effect on the insulin secretion in INS-1E cells after 24 hours stimulation (P = 0.84, n = 5). The removal of peptides <100–500 Da (by dialysis) and 3000 Da (centrifugal filtration) did not reduce the ability of gliadin digest to stimulate insulin secretion in INS-1E cells (dialysis: P = 0.026, filtration: P = 0.003, n = 3).

To address whether increased weight could result directly from beta cell exposure to gliadin digest, we stimulated INS-1E cells with 300 µg/ml gliadin digests for 24 hours. This led to a 1.8±0.3 fold increase in insulin secretion (Fig. 2A, P<0.0001, n = 6). Further, gliadin digest incubations between 30 µg/ml and 600 µg/ml yielded a dose-dependent response in INS-1E cells (Fig. 2B, P<0.0001, n = 6). Endotoxins present in the digestion enzymes (approximately 15 ng/ml in the stimulation media) were not responsible for this effect, as cells incubated with a solution of the heat-inactivated digestion enzymes or high doses of endotoxin (lipopolysaccharide 5 µg/ml) did not display increased insulin secretion (Fig. 2A). The effect of gliadin digest was independent of glucose, as stimulation in the presence of 3 mM glucose led to an increase in insulin secretion by 1.7±0.4 fold (Fig. 2C, P = 0.01, n = 4). Moreover, the increased insulin secretion was not due to gliadin-induced cell proliferation or an increased cell mass. This was confirmed by a resazurin conversion assay, which showed no difference in cell mass with or without gliadin-digest exposure (Fig. 2D). Additionally, incubation with digested ovalbumin for 24 hours did not increase insulin secretion (Fig. 2E, P = 0.02, n = 4), suggesting a specific effect for digested gliadin. A 1.6±0.4 fold increase in insulin secretion was also observed in isolated rat islets of Langerhans, following a 24 hour exposure to 300 µg gliadin digest/ml, as compared to incubation with only glucose (Fig. 2F, P = 0.015, n = 4). To identify a more specific gliadin fragment as the stimulatory factor, we tested two fragments, a 33-mer [2] and a 19-mer [16]. INS-1E cells incubated with the 33-mer for 24 hours exhibited a dose-dependent response (Fig. 2G, P = 0.03, n = 4), with up to 1.7 fold more insulin secreted at the largest tested concentration, compared to both controls and cells incubated with the 19-mer. This suggests that the 33-mer may be the fragment responsible for stimulation in gliadin digest.

### Gliadin digest has no acute effects on insulin secretion

To investigate immediate temporal insulin secretion activity, cells were challenged with gliadin digest for 30 minutes. This did not increase basal or stimulated levels of insulin secretion compared to low glucose alone (Fig. 2H, P = 0.43 and P = 0.31, n = 4). Likewise, preincubation with gliadin digest for 24 hours prior to glucose stimulation in calcium-5 buffer did not increase the glucose-induced insulin secretion (Fig. 2H, P = 0.59, n = 4).

### Low molecular weight fragments in gliadin digest do not mediate gliadin-induced insulin secretion

Amino acids can affect insulin secretion [17], [18]. To investigate whether the effect of gliadin digest was due to presence of amino acids in the digest, we incubated INS-1E cells with 1 mM arginine for 24 hours and observed no difference in insulin secretion (Fig. 2I). Furthermore, we used two methods to separate the low-molecular compounds from the gliadin solution: dialysis (MWCO 100–500 Da) and filter centrifugation (MWCO 3000 Da). Removal of these fractions did not affect the ability of gliadin digest to increase insulin secretion (Fig. 2I), which suggests that the effect is not due to low-molecular weight compounds.

### Gliadin digest-induced insulin secretion is not affected by MyD88 or FFAR1 downregulation, but gliadin digest potentiates fatty acid-induced insulin secretion

**Figure 3:**
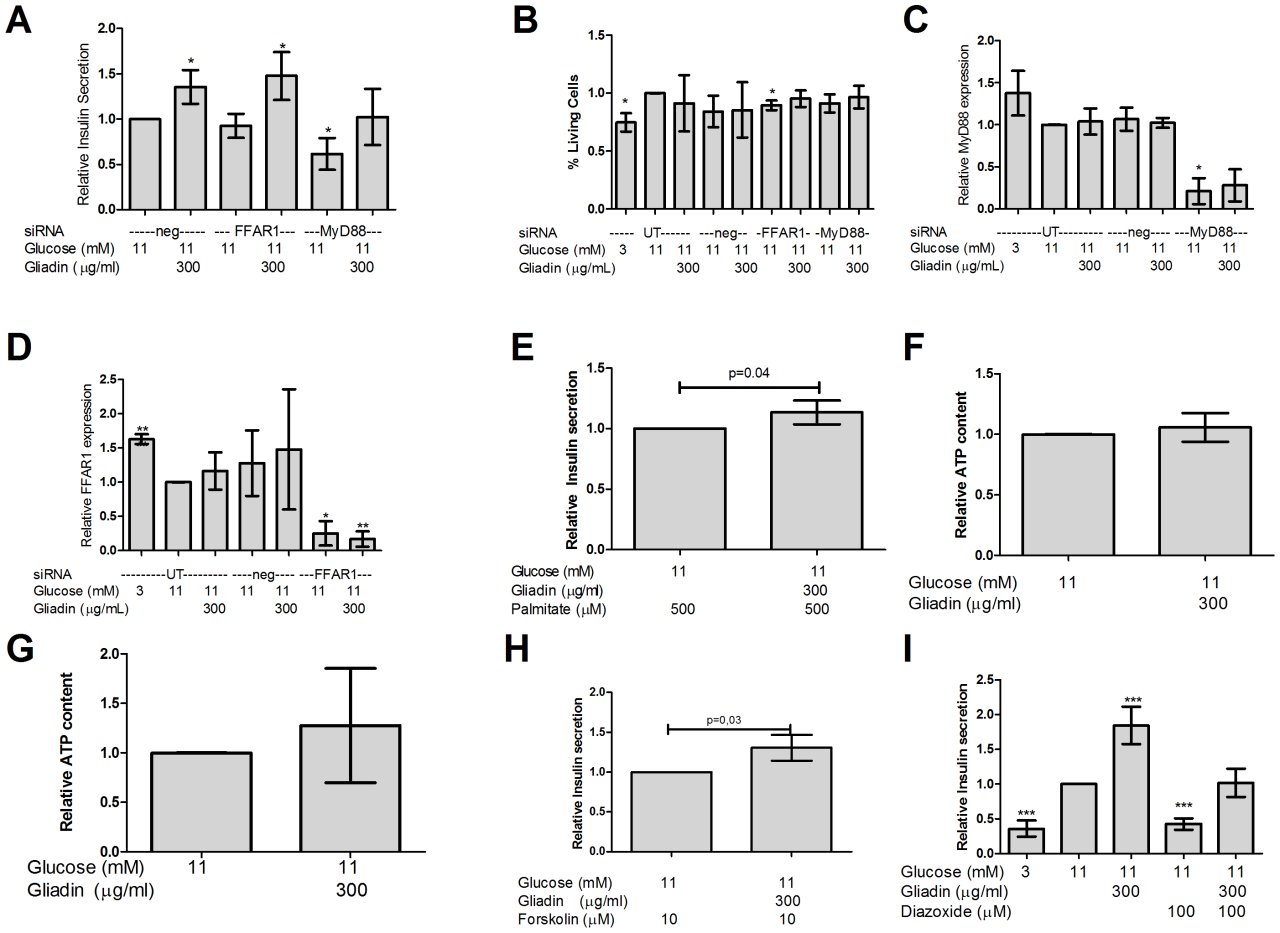
The effect of gliadin digest is independent of FFAR1 and MyD88, but is abrogated by diazoxide treatment. A. Fatty acid receptor FFAR1 downregulation using siRNA, did not affect insulin secretion during 24 hours of gliadin digest stimulation (P = 0.48, n = 4). Following MyD88 downregulation, glucose induced insulin secretion was reduced to 61% (0.6±0.3X, P = 0.02, n = 4). Though not significant, gliadin digest was still able to increase insulin secretion in the cells with downregulated MyD88 (P = 0.06, n = 4). B. No significant cell death was observed after transfections, except for cells treated with FFAR1 siRNA (P = 0.05, n = 3). C. Efficiency of the MyD88 silencing was confirmed using qPCR, which downregulated MyD88 expression as low as 25% of baseline expression levels (0.25±0.38X, P = 0.01, n = 3). D. Efficiency of the FFAR1 silencing was confirmed using qPCR, which downregulated FFAR1 expression as low as 25% of baseline expression levels (0.25±0.28x, P<0.01, n = 3). In low-glucose medium, FFAR1 expression was increased by 63% (1.63±0.17x, P<0.01, n = 4). E. Cells treated with gliadin digest for 24 hours prior to stimulation with palmitate and glucose for 30 min secreted 13% more insulin than palmitate-stimulated control cells (1.13±0.12x, P = 0.04, n = 5). F. Gliadin digest did not increase ATP content in INS-1E cells after 24 hours, as compared to controls (P = 0.34, n = 5). G. No significant increase in intracellular ATP was detected in cells stimulated with glucose and gliadin digest for 30 min compared to controls (P = 0.35, n = 4). H. Insulin secretion was increased by 31%, when cells were stimulated with both forskolin and gliadin digest compared to forskolin alone (1.31±0.26x, P = 0.03, n = 4). I. Addition of 100 µM diazoxide to medium with 11 mM glucose, reduced insulin secretion to 43% of normal secretion levels (0.43±0.13x, P = 0.0008, n = 4), similar to levels secreted in low glucose medium. However, when diazoxide was added to cells stimulated with gliadin digest and glucose, insulin secretion was restored to the levels observed for 11 mM glucose alone (P = 0.87, n = 4).

As gliadin is highly hydrophobic, it can interact with receptors recognising fatty acids or LPS, such as toll-like receptor 4 [19]. We investigated whether the effect of gliadin digest was dependent on two proteins associated with fatty acid-stimulated pathways; GPR40/FFAR1, a fatty acid receptor potentiating insulin secretion [20], and MyD88, a component in TLR2/4 signalling [21]. When FFAR1 was downregulated by siRNA, gliadin digest-induced insulin secretion was unchanged (Fig. 3A, P = 0.48, n = 4). MyD88-downregulated cells expressed trends of increased insulin secretion when stimulated with gliadin digest. However, the cells secreted significantly less insulin upon glucose stimulation, only 61±28% compared to the control group (Fig. 3A, P = 0.02, n = 4). This was not due to cell death during transfection (Fig. 3B, P = 0.5, n = 3). To confirm downregulation of the target genes, we used quantitative RT-PCR (Fig. 3C–D). In the control group receiving low glucose, we observed a 1.63±0.17 fold increased expression of FFAR1 (Fig. 3D, P<0.01, n = 4). To investigate whether cells incubated with gliadin digest exhibited an altered response to fatty acids, we stimulated cells with gliadin digest overnight. This subsequently led to a 13±12% increased insulin secretion response to fatty-acid stimulation (Fig. 3E, P = 0.04, n = 5).

### Gliadin digest-mediated insulin secretion is abrogated by diazoxide treatment, and is not due to ATP or cAMP production

To ensure that the observed increase in insulin secretion was not due to gliadin catabolism, we measured intracellular ATP levels following gliadin digest exposure. No significant increase was observed in intracellular ATP content after 24 hours or 30 min gliadin digest incubation as compared to control groups (Fig. 3F–G). To investigate if gliadin digest potentiated insulin secretion by cyclic AMP (cAMP)- production, we used the adenylate cyclase activator forskolin, to increase intracellular cAMP. During forskolin stimulation, gliadin digest could further increase insulin secretion by 31±26% compared to forskolin alone (Fig. 3H, P = 0.03, n = 4), suggesting that the effect of gliadin digest is not mediated through activation of cAMP-stimulated pathways. To address gliadin digest effects on ATP-sensitive potassium channels (K_ATP_) in the insulin secretion pathway, we used the K_ATP_ channel activator diazoxide. Co-incubation with diazoxide and gliadin digest neutralized the stimulatory effect of gliadin digest (Fig. 3I, P = 0.001, n = 4). This suggests that gliadin-induced insulin secretion is mediated through K_ATP_ channels.

### Gliadin digest incubation inhibits K_ATP_-current

**Figure 4:**
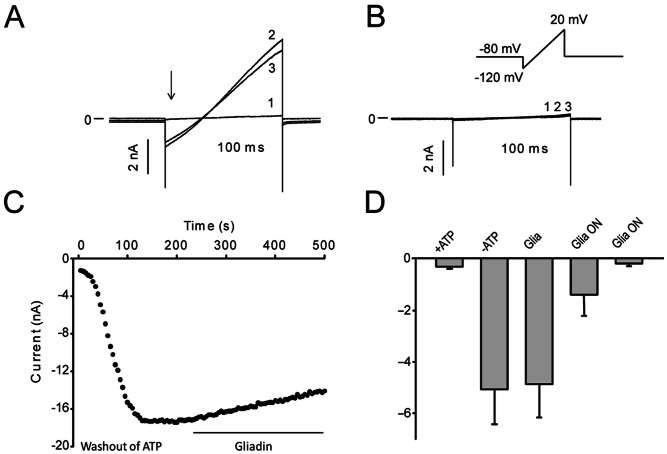
Gliadin digest incubation inhibits current through K_ATP_. K_ir_6.2 and SUR1 were expressed in HEK-293 cells. The cells were incubated overnight in either 300 µg/ml gliadin digest or a corresponding volume of enzyme mixture. Currents were activated by a ramp protocol every 5 s. A. Representative K_ATP_ currents in a cell incubated with the enzyme mixture overnight (1) prior to ATP washout, (2) after ATP washout in the presence of enzyme mix and (3) after 2.5 minutes exposure to gliadin digest. B. Representative currents (traces overlaid in plot) from a cell incubated with gliadin digest overnight, (1) prior to ATP washout, (2) after ATP washout in the presence of the enzyme mixture and (3) after application of the enzyme mixture for 2.5 minutes. C. The time-dependence of the effect of gliadin digest on K_ATP_ currents. Representative data from a cell incubated in enzyme mix overnight. The data points represent maximal inward current during washout of endogenous ATP and after application of gliadin digest. D. Summarized current densities in : +ATP, average current density prior to ATP washout in cells incubated in enzyme mix overnight (n = 7), −ATP, average current density after washout (n = 9). Gliadin digest: average current density after 2.5 min exposure to 300 µg/ml gliadin digest (n = 9). The effect of gliadin digest was not significant. In 10/12 cells incubated in gliadin digest overnight there was no expressed K_ATP_ current. In the first bar graph all cells are included; in the second bar graph the 2 cells with current are excluded. For cells incubated in the enzyme mixture, 3/12 cells had no current and were excluded from the analysis.

To examine the direct effect of gliadin digest on K_ATP_ current, we measured whole-cell current following gliadin digest stimulation. This was investigated in HEK-293 cells expressing both the K_ir_6.2 pore-forming subunits and sulfonylurea receptor 1 (SUR1) of the K_ATP_ channel. The SUR1 contains nucleotide binding domains that are critical in sensing the metabolic status of cells. Whole-cell currents were recorded using a 200 ms ramp protocol ranging from −120 mV to +20 mV. For the cells incubated in enzyme mix, the initial K_ATP_-currents were nearly absent (0–1 nA) though following dilution of endogenous ATP with the recording pipette solution, current levels increased (Fig. 4A). In cells incubated in gliadin digest for 24 hours, 10 out of 12 cells did not express K_ATP_-currents subsequent to ATP washout (Fig. 4B). Acute application of gliadin digest to the cells resulted in a 6.7±2.6% decrease in current after 2.5 min, but this change was not significant (Fig. 4C). Addition of 5 mM BaCl_2_ at the end of the experiment confirmed that the recorded current was mediated by K_ATP_ [22]. Results are summarized in Fig. 4D.

### The gliadin 33-mer reduces current through K_ATP_ channels

**Figure 5:**
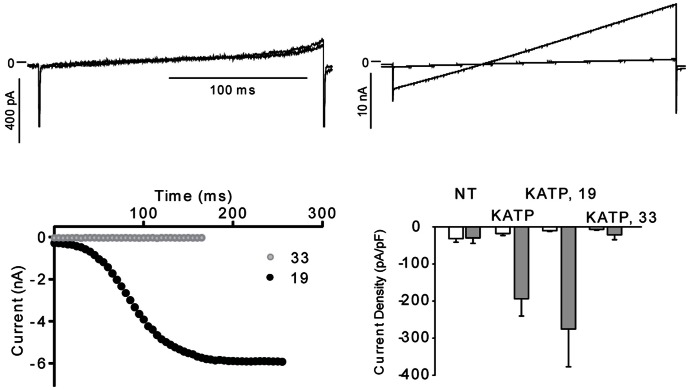
The 33-mer gliadin fragment inhibits K_ATP_ currents. K_ir_6.2 and SUR1 were transiently expressed in HEK-293 cells. The cells were incubated with either the 19-mer or 33-mer gliadin fragment in the medium overnight. Currents were activated by a ramp protocol every 5 s as described in Fig. 4. A. Representative K_ATP_ currents in a cell incubated with the 19-mer. Trace (1): prior to washout of endogenous ATP, trace (2): after washout of ATP. B. Representative currents from a cell incubated with the 33-mer before and after washout of ATP. C: The time-dependence of the effect of the 19-mer (black circles) or the 33-mer (grey circles) on maximum inward current. The data points represent maximum inward current at the start of the ramp protocol during washout of endogenous ATP. D. A summary of current densities. Non-transfected cells (NT, n = 3), transfected cells incubated in control medium (K_ATP_, n = 7), transfected cells incubated in the 19-mer (K_ATP_, 19, n = 9) or transfected cells incubated in the 33-mer (K_ATP_, 33, n = 9). Average current density before (white) and after (grey) ATP washout is shown.

To test whether the gliadin 19- or the 33-mer could affect K_ATP_ currents, K_ir_6.2 and the SUR1 were expressed in HEK-293 cells and the cells were incubated overnight in the presence of the 19- or the 33-mer. Whole-cell currents were compared to controls after washout of endogenous ATP (Fig. 5A–D). HEK-293 cells that had been exposed to 100 µM 19-mer had currents comparable to those of controls, whereas cells exposed to 100 µM 33-mer, exhibited significantly reduced currents (Fig. 5D). The 33-mer can therefore affect the K_ATP_-channel in the same manner as the gliadin digest.

## Discussion

We have demonstrated that gliadin digest potentiates insulin secretion in INS-1E cells and rat islets, independently of glucose levels. This effect relies on closure of the ATP-sensitive potassium channels. The protease-resistant 33-mer gliadin fragment, which is generated in large quantities by enzymatic digestion of gliadin [2], may be the stimulatory component in the gliadin digest. Due to the observed weight gain in NOD mice following gliadin digest administration, we found it reasonable to investigate the effect of gliadin *in vitro* on beta cells. We hypothesised the increased weight was a consequence of increased insulin secretion, which induced a trophic effect in the insulin target tissues. Nevertheless, the observed mechanism remains unproven *in vivo*. Furthermore, gliadin digest injections did not accelerate the development of diabetes in NOD mice. This results is corroborated in parallel by the finding that a gluten enriched diet does not increase NOD diabetes incidence [7].

Although intravenous injection of gliadin fragments is not physiological, data suggest that undigested gliadin fragments do cross the intestinal barrier *in vivo*. *First*, gliadin fragments have previously been demonstrated in breast milk, which alludes to its passage through healthy gut epithelium in patients with and without celiac disease [4]. *Second*, the 33-mer is transported across Caco-2 colon carcinoma cells in an un-cleaved form via transcytose [23], a process which is stimulated by interferon gamma. The 33-mer was shown to be transported into the early endosomes of duodenal biopsies from patients with active celiac disease, but it was not found to associate with the late endosomes. This suggests that the fragments escape lysosomal degradation [3]. *Third*, gliadin induces zonulin release in intestinal epithelial cell lines, resulting in increased monolayer permeability [24]. This indicates that transport may occur through and between the intestinal cells. *Fourth*, increased intestinal permeability has been described in patients with type 1 diabetes [5]. Moreover, BB rats also have increased intestinall permeability, as they have reduced expression of the tight junction protein claudin-1 as compared to the Wistar rat, which correlates to increased intestinal permeability [25]. Additionally, infection with enterovirus has been shown to increase intestinal permeability [26], which is of particular interest in T1D. Enterovirus infection is frequently associated with the development of type 1 diabetes [27]. Hence, increased intestinal permeability may provide a mechanism for gliadin entry into the bloodstream of diabetes patients, We therefore propose that diabetes patients may be exposed to increased levels of gliadin peptides, due to the above mentioned factors. Further, it is possible that transepithelial transport of fragments may not be correlated to permeability and “leakiness”, but is a specific process, as was recently described in patients with active celiac disease [3].

Islets in the prediabetic mice may experience heightened exposure to gliadin, due to an increase in vascular endothelium permeability. A study reporting diffusion of a 70 kDa dye from the vascular confinement of the islets into the surrounding acinar tissue illustrated altered vascular permeability in prediabetic mice [28]. This was not observed in healthy mice and would suggest that in prediabetic mice, increased endothelial permeability may potentially facilitate increased entry of gliadin fragments into the islets. Similarly, in both diabetes-prone and diabetes-resistant BB rats, endothelial permeability was higher in the pancreatic venules. This was visualised by injection with Monastral Blue B dye [29], and permeability was compared to three different control rat strains. Finally, in female NOD mice, increased blood flow was detected through the islets at 10 to 14 weeks of age, as compared to males of similar age and female ICR mice [30]. This process was mediated by excessive nitric oxide production [30], and it may increase exposure of the beta cells to gliadin fragments. Although likely mediated by ongoing inflammation, it is feasible that gliadin fragments in contact with beta cells may activate the cells by the mechanism described in this study.

Observations implicate gluten's influences on the development of diabetes in humans. A particular compelling T1D case describes a patient, who remained healthy, devoid of insulin therapy for 36 months, after being prescribed a gluten-free diet at the time of T1D diagnosis. His stable fasting blood glucose levels at 5.8 mmol/l, indicate that a gluten-free diet may be a method to protect beta-cell function in patients. However, the protective effect remains to be tested in clinical trials of T1D patients [9]. This gluten-free diet approach may enhance beta-cell rest, by circumventing gliadin-induced increase in beta-cell activity, which we have described in this study. A study of T1D patients' realatives exhibited an increased acute insulin response subsequent to 6 months on a gluten-free diet. The improved insulin response dissipated upon reintroduction of gluten into their diet [31]. This suggests that the cells are more active in patients on a gluten-containing diet. Thus, gluten peptides lower the insulin response to glucose over time, or contribute to a higher insulin secretion at basal glucose levels. If gliadin contributes to beta-cell activity *in vivo*, this may likely impact the development of diabetes development. Increased insulin secretion correlates to increased diabetes development (reviewed in [32] and [1]) and first-degree relatives to T1D patients have increased levels of insulin in the blood [33]. In patients, induced beta-cell rest is beneficial for preservation of beta-cell function [34], and several suggestions have been made for this association. These include decreased surface expression of beta-cell antigens, reduced sensitivity towards cytokines and changes in gene expression (summarized in [1]). As many people consume gluten throughout the day on a standard western diet, cells may be continuously exposed to gliadin fragments, affecting the cells' insulin secretion. However, other wheat proteins likely contribute to diabetes development apart from gliadin, as a gluten-free diet supplemented with gliadin only partially restores incidence of diabetes in NOD mice [35].

This observed insulin secretion effect hints that a gluten-free diet may also be beneficial in preserving beta-cell function in type 2 diabetes (T2D). However, a study of T2D patients did not identify any alterations in intestinal permeability [36], meaning that exposure to gliadin is not likely increased under normal conditions. On the other hand, this does not exclude a role for gliadin since changes in permeability may occur transiently or as a specific response to gliadin fragments, as seen in celiac disease [37]. A high-fat diet has been shown to increase intestinal permeability, likely due to a reduction in the expression of ZO-1 and Occludin protein [38].

Our finding that treatment of INS-1E cells with gliadin digest exhibits increased insulin secretion upon palmitate challenge, shows that gliadin digest primes beta cells to react more strongly to fatty acids. Beta-cell function in type 2 diabetes improves significantly in response to induced beta-cell rest, illustrated by diazoxide [39]. As gliadin and fatty acids are often consumed together, gliadin fragments may decrease beta-cell rest if a high fat diet is consumed. A recent case study, describing an overweight 51-year old woman with iron deficiency anaemia reported resolution of metabolic syndrome in the patient, following six months on a gluten-free diet [40]. This suggests that gluten may be a contributing factor in the development of metabolic syndrome.

Neither FFAR1, nor MyD88 signalling were identified as gliadin-activated targets, but both genes yielded intriguing observations. Surprisingly, our findings show that MyD88 is required for a normal insulin response to glucose in INS-1E cells, which suggests a novel role for this protein. This is in contrast to a previous study, showing that islets from MyD88 -/- mice respond to glucose similarly to wild-type mice. However, these mice became more susceptible to hyperglycaemia in prediabetic conditions [41].

In conclusion, our findings indicate that gliadin components, particularly the gliadin 33-mer, induce insulin secretion in INS-1E cells and rat islets of Langerhans. This study describes a potential mechanism for substantiating the beneficial effect of a gluten-free diet on T1D.
